# Thoracolumbar Rotation During Tai Chi Movements—A Biomechanical Analysis of the Entire Peking Style Routine

**DOI:** 10.3389/fspor.2022.834355

**Published:** 2022-02-02

**Authors:** Christian Wehner, Carina Wehner, Hermann Schwameder, Wolfgang Schobersberger

**Affiliations:** ^1^Institute for Sports Medicine, Alpine Medicine and Health Tourism, UMIT – Private University for Health Sciences, Medical Informatics and Technology, Hall in Tirol, Austria; ^2^WuYuan – School of Chinese Martial Arts, Kungfu, Taijiquan and Qigong, Munich, Germany; ^3^Department of Sport and Exercise Science, University of Salzburg, Salzburg, Austria

**Keywords:** Tai Chi, biomechanics, spine, flexibility, thoracolumbar rotation, postural balance

## Abstract

The impact of spinal mobility and sagittal spinal shape on the development of balance impairment supports the hypothesis that enhancing spine flexibility results in an improvement in postural balance ability. Therefore, the aim of this study was to investigate whether the range of motion of thoracolumbar rotation during the movements of the Tai Chi Peking style routine is sufficient to improve thoracolumbar flexibility. Three-dimensional kinematic and kinetic data were collected from eight athletes of the German Wushu Federation, while performing all movements of the entire Peking style routine (1) in a competition version corresponding to national/international championships and (2) in a health sport version performed with shorter and higher stances (i.e. a smaller distance between the feet and thus less knee flexion). For each movement the total mean and standard deviation values for the total range of motion of thoracolumbar rotation was calculated. Statistical analysis was performed using the Wilcoxon signed-rank test for paired differences. Eight movements showed major differences (10.12–19.73°) between the two versions. For the remaining movements, only minor differences (0.7–9.56°) were observed. All movements performed on both sides showed no significant lateral differences. Most of the Tai Chi movements, regardless of the performed version, cover a range of motion of thoracolumbar rotation that has the potential to lead to an improvement of thoracolumbal spine flexibility with appropriate training. The most effective single movements (25.97–72.22°) are *Brush Knee and Step Forward, Step Back and Repulse Monkey, Grasp the Sparrow‘s Tail, Wave Hand in the Clouds*, and *Fair Lady Weaves with Shuttle*.

## Introduction

Tai Chi Quan, often just called Tai Chi, is widely known as a healthy form of exercise, especially for older adults and individuals with chronic diseases (Hempel et al., 2014; Chen et al., 2016). As a style of Chinese martial arts (Wushu), Tai Chi includes defined motion sequences performed with and without weapons (e.g. swords), as well as routines performed with partners. Chen, Yang, Wu Yuxiang, Wu and Sun style are the five traditional styles of Tai Chi. The so-called Peking style routine, which was published in the 1950s and features 24 movements based on the Yang style, is the most famous routine in the field of health sports (Guo et al., 2014).

In the past few decades, a considerable number of studies, systematic reviews and meta-analyses concerning Tai Chi have been published. Most of them deal with health aspects of Tai Chi, e.g. its effects on individuals with chronic obstructive pulmonary disease (COPD) (Chen et al., 2016; Wang et al., 2019), type 2 diabetes mellitus (Zhou et al., 2019), coronary heart disease (Liu et al., 2018) or its ability to improve balance and mobility in individuals with Parkinson disease (Hackney and Earhart, 2008). A meta-analysis examining the effect of Tai Chi training on muscle strength, physical endurance, postural balance and thoracolumbal spine flexibility revealed significant enhancement of the standing time in single-leg-stance with open eyes and of thoracolumbal spine flexibility (Wehner et al., 2021). When analyzing the large heterogeneity via univariate meta-regression analysis regarding thoracolumbal spine flexibility, 91.4% of the between study heterogeneity could be explained by the existence of a balance impairment (Wehner et al., 2021). This is most apparent in the study of Zhang et al. (2006), who investigated the influence of Tai Chi on balance-impaired participants with a higher risk of falling (defined by a one leg standing time between 5 and 20 s). Significant improvements in both thoracolumbal spine flexibility (+4.5 cm) and the single-leg-stance test with open eyes (+12.3 s) have been reported. According to Kasukawa et al. (2010) and Ishikawa et al. (2017), spinal mobility and sagittal spinal shape are causal factors with respect to displacement of the center of gravity, which results in balance impairment. This relation supports the hypothesis that enhancing thoracolumbal spine flexibility leads to an improvement in postural balance ability (Wehner et al., 2021).

A question that emerges in this context is whether the performance of the movements is also an important aspect. Lan et al. (1998) suggest that the physical impact of Tai Chi depends on the execution of its movements. Similarly, Wen et al. (2018) report a difference in lower vs. higher stances (poses) during the execution of Tai Chi movements. Therefore the aim of this study was not only to test the hypothesis that the movements are large enough to have a possible impact on spine flexibility, but also to compare two different types of performance (health and competition style) of the Tai Chi movements. Furthermore, lateral differences in movements executed in both directions should be analyzed. As in some studies (Gatts, 2008), only a small number of Tai Chi movements out of a complete routine (e.g. Peking style routine) were used, another aim was to determine the movements with the greatest potential for postural balance enhancement. Therefore all movements of the entire Peking style routine were investigated.

To address the first objective, thoracolumbar spine movement was investigated during the complete Tai Chi Peking style routine. In this study the analysis focused on thoracolumbar rotation, as (1) Tai Chi movements are mainly performed in an upright position without spinal movements in the frontal or sagittal plane and (2) thoracolumbar rotation is important for the typical flowing Tai Chi motions. Furthermore we examined the magnitude of the difference between the health sport version (HS), which is commonly used for daily practice, and the competition version (CV), which involves the highest level of physical exertion. Moreover we examined the lateral differences in movements executed in both directions.

## Materials and Methods

To investigate our objectives, we conducted a biomechanical analysis in a group of eight athletes (males and females), each performing both versions of the entire Peking style routine directly after each other.

### Participants

Highly experienced athletes from the German Wushu Federation were invited to participate. The participants had to fulfill the following criteria: aged 18 years or above, at least 5 years of training experience in Tai Chi, and at least fifth place at the German Tai Chi championship. Exclusion criteria were injuries, illnesses or health conditions that preclude participation in sporting activities, and allergies to adhesives (because of the use of reflective markers for the biomechanical analysis).

After ethical approval was obtained, the athletes were recruited by email and signed a written informed consent.

Three healthy males and five healthy females (age: 44 ± 8 years, height: 1.70 ± 0.07 m, body mass: 65.2 ± 11.7 kg) participated in this study. Training experience ranged from 6 to 30 years. All athletes achieved first place at the German Tai Chi championship at least once. One participant ranked first, and another sixth, at the European Tai Chi championships, and one athlete ranked sixth at the Tai Chi World championships. Except for one who had not graduated, all athletes had a graduation level of 3–6 in Tai Chi according to the Chinese Wushu Duanwei System. The characteristics of the participants are shown in Table 1.

**Table 1:** Characteristics of the participants.

**Athlete**	**Gender**	**Age (y)**	**Body height (m)**	**Body weight (kg)**	**Training experience (y)**	**Duan level**
1	f	36	1.62	57.8	6	3
2	f	36	1.70	64.2	19	3
3	f	47	1.58	48.9	12	3
4	m	48	1.70	66.9	18	5
5	m	31	1.82	88.0	15	0
6	m	44	1.75	73.6	19	3
7	f	52	1.68	58.9	9	3
8	f	54	1.72	63.0	30	6

*m/f, male/female; y, years*.

### Instrument

Three-dimensional kinematic and kinetic data were collected at 250 Hz and 1,000 Hz, respectively, using a 13-camera motion capture system (Qualisys AB, Gothenburg, Sweden) and two integrated force plates (60 × 120 cm; AMTI, Watertown, MA, USA). In total 57 reflective markers were placed bilaterally to participants head, upper body, upper limbs, and lower limbs, based on the Cleveland Clinic Marker Set (Motion Analysis Corp, Santa Rosa, CA, USA), which uses an anatomical landmark calibration technique.

### Experimental Protocol

**Figure 1:**
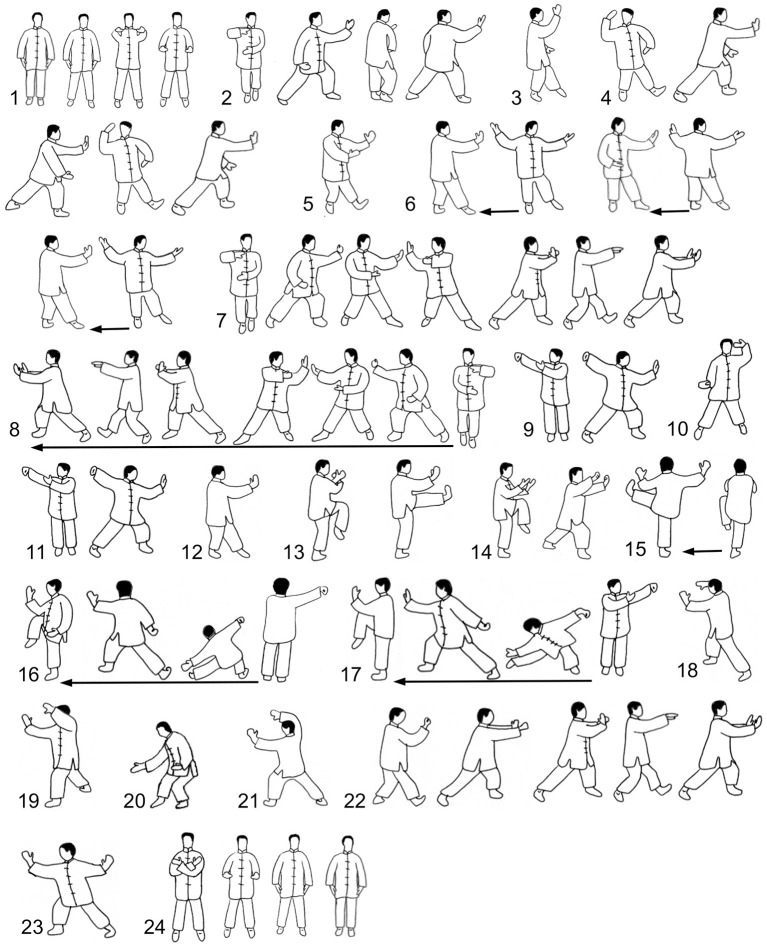
Movements of the Peking style routine. 1, Beginning; 2, Part the wild horse's mane (left and right); 3, White crane spreads its wings; 4, Brush knee and step forward (left and right); 5, Playing the lute; 6, Step back and repulse monkey (left and right); 7, Grasp the sparrow's tail (left); 8, Grasp the sparrow's tail (right); 9, Single whip; 10, Wave hand in the clouds; 11, Single whip; 12, High pat on horse; 13, Right heel kick; 14, Strike to ears with both fists; 15, Left heel kick; 16, Lower body and stand on one leg (left); 17, Lower body and stand on one leg (right); 18, Fair lady weaves with shuttle (right and left); 19, Pick up the needle from the sea bottom; 20, Fan back; 21, Turn body, Deflect, Parry, and Punch; 22, Seal tightly; 23, Cross hands; and 24, Closing.

**Figure 2:**
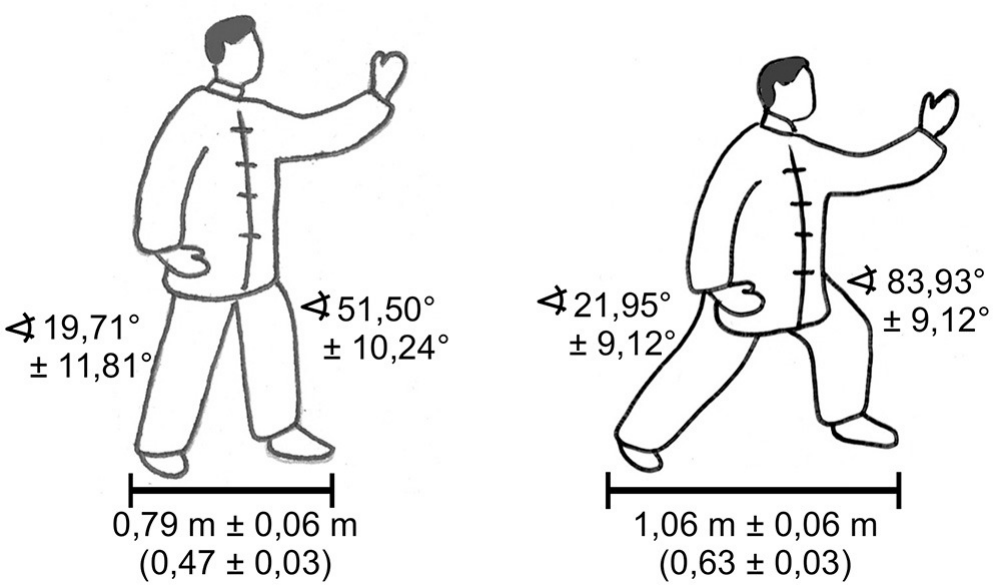
The length and height of the stances according to knee angles and stance length in the bow stance (Gong Bu).

After a 15–20-min individual warm-up, each participant was equipped with reflective markers. Subsequently, each participant performed a 10 m-walk over the two force plates to calibrate the individual measuring instruments. Since only two force plates were available, the whole Peking style routine (shown in Figure 1) had to be separated into single movements. Moreover, repetitive movements were performed only once. Each participant performed all 24 movements twice: first as the CV (corresponding to national/international championships) and second, after a short break, as the HS (with shorter and higher stances, that means a smaller distance between the feet and thus less knee flexion). To define the length and height of the stances, as well as the range of motion of the upper body and upper limbs, the bow stance (Gong Bu) taken in the movement called *Part the Wild Horse‘s Mane* was used. All other movements were adjusted according to this movement. The average dimensions for the two versions are shown in Figure 2.

### Data and Statistical Analysis

All raw kinematic and kinetic data were labeled and cut using QTM (Qualisys, Göteborg, Sweden). Further analysis was performed using Visual3D (C-Motion Inc., Germantown, MD, USA). Raw kinematic data were filtered with a 15 Hz Butterworth filter, and raw kinetic data with a 25 Hz Butterworth filter. For the statistical analysis, IBM SSPS Statistics 27 (IBM Corp., Armonk, NY, USA) was used. Thoracolumbar rotation was calculated from the torsion of shoulder axis and pelvis. For the single movements, the mean and standard deviation values for thoracolumbar rotation to the left and right were calculated, and the total range of motion of the thoracolumbar rotation was determined. The range of motion of the CV and HS were compared using the Wilcoxon signed-rank test for paired differences. Movements performed on both sides were also compared using the Wilcoxon signed-rank test for paired differences, to examine side differences within the same performance (CV or HS). Differences were considered significant at *p* < 0.05. In order to determine which movements comprised large thoracolumbar rotation, the range of motion was compared with thoracolumbar rotation during the 10 m-walk at the beginning of the trial, which revealed a mean thoracolumbar rotation of 20.0° ± 7.7°.

## Results

### Health Sport Version

The means and standard deviations of thoracolumbar rotation for all movements are shown in Table 2. In total, 18 out of 27 movements have a range of motion larger than 20°: *White Crane Spreads Its Wings, Brush Knee and Step Forward (right), Step Back and Repulse Monkey (left), Step Back and Repulse Monkey (right), Grasp the Sparrow‘s Tail (left), Grasp the Sparrow‘s Tail (right), Wave Hand in the Clouds, Strike to Ears with Both Fists, Left Heel Kick, Lower Body and Stand on One Leg (left), Lower Body and Stand on One Leg (right)*, and *Seal Tightly*. Five of them have a range of motion at least twice as large as 20° (i.e. > 40°): *Brush Knee and Step Forward (left), High Pat on Horse, Fair Lady Weaves with Shuttle (right), Fair Lady Weaves with Shuttle (left)*, and *Pick Up the Needle from the Sea Bottom*.

**Table 2:** Range of motion of thoracolumbar rotation.

**Movement name**	**HS**	**CV**	**Diff**	***p***
Beginning	4.70 ± 1.23	5.47 ± 1.27	0.77	0.036
Part wild horse‘s mane (left)	14.68 ± 4.17	21.27 ± 8.73	6.59	0.025
Part wild horse‘s mane (right)	14.51 ± 5.17	21.23 ± 10.82	6.72	0.069
White crane spreads its wings	27.00 ± 10.37	39.38 ± 14.91	12.38	0.025
Brush knee, step forward (left)	55.04 ± 12.09	51.85 ± 7.97	3.19	0.327
Brush knee, step forward (right)	34.97 ± 6.12	46.60 ± 9.50	11.63	0.012
Playing the lute	20.89 ± 5.44	27.04 ± 6.96	6.15	0.050
Step back, repulse monkey (left)	32.54 ± 8.45	31.81 ± 5,98	0.73	0.575
Step back, repulse monkey (right)	25.97 ± 6.56	27.55 ± 6.92	1.58	0.327
Grasp the sparrow‘s tail (left)	31.78 ± 9.09	44.65 ± 8.21	12.87	0.012
Grasp the sparrow‘s tail (right)	33.42 ± 11.26	45.42 ± 13.84	12.00	0.017
Single whip	12.72 ± 4.49	21.46 ± 10.87	8.74	0.017
Wave hand in the clouds	38.28 ± 4.95	42.61 ± 7.13	4.33	0.025
High pat on horse	45.05 ± 6.49	44.57 ± 6.55	0.48	0.889
Right heel kick	15.32 ± 5.01	24.88 ± 7.84	9.56	0.012
Strike to ears with both fists	30.37 ± 4.13	42.73 ± 7.81	12.36	0.012
Left heel kick	25.45 ± 9.98	33.70 ± 9.96	8.25	0.050
Lower body, stand on one leg (left)	30.73 ± 4.52	34.57 ± 4.26	3.84	0.123
Lower body, stand on one leg (right)	31.68 ± 4.62	36.24 ± 10.21	4.56	0.327
Fair lady weaves with shuttle (right)	44.09 ± 11.97	59.26 ± 9.35	15.17	0.017
Fair lady weaves with shuttle (left)	52.49 ± 10.22	72.22 ± 9.95	19.73	0.012
Pick up the needle from the sea bottom	48.22 ± 7.99	58.34 ± 10.01	10.12	0.012
Fan back	9.82 ± 5.28	18.13 ± 6.70	8.31	0.017
Turn body, deflect, parry, and punch	17.44 ± 6.11	18.27 ± 7.21	0.83	0.674
Seal tightly	31.97 ± 9.86	35.32 ± 8.72	3.35	0.017
Cross hands	13.14 ± 6.52	17.44 ± 8.58	4.30	0.025
Closing	11.05 ± 5.98	18.16 ± 12.89	7.11	0.093

*Values are mean ± standard deviation degrees. HS, health sport version; CV, competition version. P-values, asymptotic significance levels (two-sided test)*.

Seven movements of the Peking style routine are performed on both sides: *Part Wild Horse's Mane, Brush Knee and Step Forward, Step Back and Repulse Monkey, Grasp the Sparrow's Tail, Heel Kick, Lower Body and Stand on One Leg*, and *Fair Lady Weaves with Shuttle*. No significant lateral differences (between the left and right sides) were observed for any of these movements (Table 3), with the exception of *Brush Knee and Step Forward* (difference: 20.1° ± 4.8°) and *Fair Lady Weaves with Shuttle* (difference: 8.4° ± 5.6°).

**Table 3:** Comparison of left and right thoracolumbar rotation (HS).

**Movement name**	**left**	**right**	**Diff**	***p***
Part wild horse‘s mane	14.68 ± 4.17	14.51 ± 5.17	0.17	1.000
Brush knee, step forward	55.04 ± 12.09	34.97 ± 6.12	20.07	0.017
Step back, repulse monkey	32.54 ± 8.45	25.97 ± 6.56	6.57	0.069
Grasp the sparrow‘s tail	31.78 ± 9.09	33.42 ± 11.26	1.64	1.000
Heel kick	25.45 ± 9.98	15.32 ± 5.01	10.13	0.050
Lower body, stand on one leg	30.73 ± 4.52	31.68 ± 4.62	0.95	0.575
Fair lady weaves with shuttle	52.49 ± 10.22	44.09 ± 11.97	8.40	0.012

*Values are mean ± standard deviation degrees. HS, health sport version; CV, competition version. P-values, asymptotic significance levels (two-sided test)*.

### Competition Version

The means and standard deviations of thoracolumbar rotation for all movements are shown in Table 2. In total, 5 of 27 movements have a range of motion lower than 20°: *Beginning, Fan Back, Turn Body, Deflect, Parry, and Punch, Cross Hands*, and *Closing*. Ten movements have a range of motion larger than 40°: *Brush Knee and Step Forward (left), Brush Knee and Step Forward (right), Grasp the Sparrow‘s Tail (left), Grasp the Sparrow‘s Tail (right), Wave Hand in the Clouds, High Pat on Horse, Strike to Ears with Both Fists, Fair Lady Weaves with Shuttle (right), Fair Lady Weaves with Shuttle (left)*, and *Pick Up the Needle from the Sea Bottom*.

No significant lateral differences were detected for any of these movements (Table 4), except for *Fair Lady Weaves with Shuttle* (difference: 13.0° ± 4.8°).

**Table 4:** Comparison of left and right thoracolumbar rotation (CV).

**Movement name**	**left**	**right**	**Diff**	***P***
Part wild horse‘s mane	21.27 ± 8.73	21.23 ± 10.82	0.04	0.889
Brush knee, step forward	51.85 ± 7.97	46.60 ± 9.50	5.25	0.327
Step back, repulse monkey	31.81 ± 5, 98	27.55 ± 6.92	4.26	0.069
Grasp the sparrow‘s tail	44.65 ± 8.21	45.42 ± 13.84	0.77	0.575
Heel kick	33.70 ± 9.96	24.88 ± 7.84	8.82	0.069
Lower body, stand on one leg	34.57 ± 4.26	36.24 ± 10.21	1.67	0.889
Fair lady weaves with shuttle	72.22 ± 9.95	59.26 ± 9.35	12.96	0.012

*Values are mean ± standard deviation degrees. HS, health sport version; CV, competition version. P-values, asymptotic significance levels (two-sided test)*.

### Comparison Between the Two Versions

Comparison of the two versions revealed a significant difference in 16 of the 27 movements (Table 2). However, the differences in the range of motion for most of the movements relative to normal thoracolumbar rotation were relatively small. The largest differences ranged between 10.1° [*Pick Up the Needle from the Sea Bottom*) and 19.7° (*Fair Lady Weaves with Shuttle (left)*], for the following eight movements: *White Crane Spreads Its Wings, Brush Knee and Step Forward (right), Grasp the Sparrow‘s Tail (left), Grasp the Sparrow‘s Tail (right), Strike to Ears with Both Fists, Fair Lady Weaves with Shuttle (right), Fair Lady Weaves with Shuttle (left)*, and *Pick Up the Needle from the Sea Bottom*.

### Summary of Results

Five movements in the HS and 10 in the CV have a range of motion exceeding 40°. These five movements in the HS, which are also in the CV, comprise *Brush Knee and Step Forward (left), High Pat on Horse, Fair Lady Weaves with Shuttle (right), Fair Lady Weaves with Shuttle (left)*, and *Pick Up the Needle from the Sea Bottom*. The additional five movements in the CV are *Brush Knee and Step Forward (right), Grasp the Sparrow‘s Tail (left), Grasp the Sparrow‘s Tail (right), Wave Hand in the Clouds*, and *Strike to Ears with Both Fists*. Comparing the HS and CV, eight movements were found with notable differences between 10.1° and 19.7°. For the remaining movements, only small differences were observed. The seven movements performed on both sides showed no significant lateral differences in either the HS or CV, except for *Brush Knee and Step Forward* and *Fair Lady Weaves with Shuttle*.

## Discussion

As Tai Chi movements are mainly performed in an upright position, the purpose of this study was to determine the amount of thoracolumbar rotation associated with the 24 movements of the entire Tai Chi Peking style routine; to the best of our knowledge, this has not been evaluated previously.

### Impact of Thoracolumbar Rotation

According to Aumüller et al. (2010), the maximum thoracolumbar range of motion is about 100°. Therefore, the following degrees of rotation correspond to percentage values. Thirteen movements of the HS are performed with a rotation between 20° and 40°, and five movements range between 40° and 55°. For the CV, most movements show a rotation above 20°, and 10 movements even exceed 40°. However, this version is typically used by athletes only.

In the study of Schenkman et al. (1997), which examined thoracolumbar rotation in four subgroups (healthy young, healthy elders, functionally limited elders, and individuals with Parkinson's disease), the largest thoracolumbar rotation measured in the functionally limited elders (49.6°) and Parkinson's disease (55.5°) subgroups was similar to the range of motion values for the five HS movements mentioned above (ranging from 44.1° to 55.0°). For the healthy young and healthy elders subgroups, the differences from the measured thoracolumbar rotation (87.6° and 70.8°, respectively) were larger, but the movements reached about 50% of the maximum rotation. Based on these values, the following five single movements, which are all performed on both sides, can be used for improving thoracolumbar rotation in cases where single movements are practiced instead of the entire Peking style routine: *Brush Knee and Step Forward, Step Back and Repulse Monkey, Grasp the Sparrow‘s Tail, Wave Hand in the Clouds*, and *Fair Lady Weaves with Shuttle*. There are three movements with considerable thoracolumbar rotation, but they have distinct disadvantages that may make them unsuitable: *High Pat on Horse* can hardly be practiced as a single movement, and *Pick Up the Needle from the Sea Bottom* and *Lower Body and Stand in One Leg* may be difficult to perform for individuals with limited mobility.

### Comparison Between the HS and CV

Wen et al. (2018) reported 58.9% greater knee flexion range of motion and a 40.2% higher peek knee extension moment when performing the bow stance in a lower rather than higher position. Since the upper limb and upper body movements are proportional to the movements of the lower limbs, we expected a large difference in the range of motion of thoracolumbar rotation between the HS and CV; surprisingly, however, the difference was not very large. Although there was a significant difference for 16 movements, half of them showed only minor numerical differences (in the single-digit range), which can be considered negligible with respect to a the maximal thoracolumbar rotation of 100° (Aumüller et al., 2010). It can be concluded that Tai Chi movements have a considerable effect on thoracolumbar rotation, even if the movements are performed with shorter and higher positions.

### Comparison Between Left and Right

In two of the seven movements of the Peking style routine performed on both sides, a lateral difference (between left and right) was observed: *Brush Knee and Step Forward* and F*air Lady Weaves with Shuttle*. In the first case, the difference can be explained by the fact that the movement started from the end of the previous movement, *White Crane Spreads Its Wings*, necessitating a rotational movement. In the second case, the opposite occurred: the necessary rotation starting from the end of the previous movement, *Stand on One Leg (right)*, up to *Fair Lady Weaves with Shuttle (right)* was smaller than the rotation needed between *Fair Lady Weaves with Shuttle (right)* and *Fair Lady Weaves with Shuttle (left)*. If this is taken in consideration, the lateral difference for these two movements disappears.

### Strengths and Limitations

This biomechanical study is the first to investigate the biomechanical characteristics of all 24 movements of the complete Peking style routine. Therefore, the main strength of this study is that all common Tai Chi movements were analyzed in terms of thoracolumbar rotation.

All movements were performed by athletes with several years of experience in the practice of Tai Chi, and thus with high proficiency. This strength, however, could also constitute a limitation, because the athletes are not used to performing movements with shorter and higher stances, as required for the HS. Thus, a third version with even shorter and higher stances, as performed by unfit older people and individuals with chronic diseases, was not conducted. It must also be taken into account that the study included a very small group of athletes.

A further potential limitation is the fact that the Peking style routine had to be separated into single movements, because only two force plates were available. However, the movements were executed highly reliably, and in a very similar manner to the sequence of the Peking style routine.

Another important aspect to consider is that this is solely a biomechanical study, investigating whether the range of motion of thoracolumbar rotation during Tai Chi movements is large enough at all to have the potential to improve the spine flexibility. Due to the nature of the study factors such as training duration and training frequency that are relevant for improving physical fitness as they are considered in intervention studies, were not taken into account. Therefore based on this study, further intervention studies need to be conducted to directly investigate the potential of Tai Chi practice to increase spinal flexibility.

## Conclusion

As most Tai Chi movements have a greater range of motion than those occurring during walking, it can be inferred that Tai Chi should be more effective for improving thoracolumbar rotation than walking. Based on these results, the possible relation between improved spine flexibility, in this case thoracolumbar rotation, and an increase of balance ability can be explained. Both, the entire Peking style routine and single Tai Chi movements can be recommended for exercise. The most effective single movements are *Brush Knee and Step Forward, Step Back and Repulse Monkey, Grasp the Sparrow‘s Tail, Wave Hand in the Clouds*, and *Fair Lady Weaves with Shuttle*.

The observed differences between the HS und CV movements were mostly significant but not relevant, considering that the maximum range of motion of thoracolumbar rotation is about 100°, and given the small differences in range of motion. Therefore, Tai Chi movements with longer and lower as well as with shorter and higher stances can be recommended for improving the range of motion of thoracolumbar rotation.

## Data Availability Statement

The original contributions presented in the study are included in the article/Supplementary Material, further inquiries can be directed to the corresponding author.

## Ethics Statement

Ethical approval was obtained by the Research Committee for Scientific Ethical Questions (RCSEQ) of UMIT-Private University for Health Sciences, Medical Informatics and Technology, Hall in Tirol. All participants signed a written informed consent to participate in this study.

## Author Contributions

ChW designed the study, supervised the data collection and analyzed the data. CaW assisted with the process of breaking down the Peking style routine into single movements, and also contributed to the data collection. HS had an advisory role. The article was written by ChW, with revisions suggested by CaW, HS, and WS. The illustrations were created by ChW. All authors contributed to the article and approved the submitted version.

## Conflict of Interest

The authors declare that the research was conducted in the absence of any commercial or financial relationships that could be construed as a potential conflict of interest.

## Publisher's Note

All claims expressed in this article are solely those of the authors and do not necessarily represent those of their affiliated organizations, or those of the publisher, the editors and the reviewers. Any product that may be evaluated in this article, or claim that may be made by its manufacturer, is not guaranteed or endorsed by the publisher.

## References

[B1] Aumüller G. Aust G. Doll A. Engele J. Kirsch J. Mense S.. (2010). Anatomie. 2. überarbeitete Auflage. Stuttgart: Thieme Verlag.

[B2] Chen Y. W. Hunt M. A. Campbell K. L. Peill K. Reid W. D. (2016). The effect of Tai Chi on four chronic conditions - cancer, osteoarthritis, heart failure and chronic obstructive pulmonary disease: a systematic review and meta-analyses. Br. J. Sports Med. 50, 397–407. 10.1136/bjsports-2014-094388 26383108

[B3] Gatts S.. (2008). Neural mechanisms underlying balance control in Tai Chi. Med. Sport Sci. 52, 87–103. 10.1159/000134289 18487889

[B4] Guo Y. Qiu P. Liu T. (2014). Tai Ji Quan: an overview of its history, health benefits, and cultural value. Journal of Sport and Health Science 3:3–8. 10.1016/j.jshs.2013.10.004

[B5] Hackney M. E. Earhart G. M. (2008). Tai Chi improves balance and mobility in people with Parkinson disease. Gait Posture 28, 456–460. 10.1016/j.gaitpost.2008.02.005 18378456 PMC2552999

[B6] Hempel S. Taylor S. L. Solloway M. Miake-Lye I. M. Beroes J. M. Shanman R.. (2014). Available online at: https://www.ncbi.nlm.nih.gov/books/n/vataichi/pdf/ (accessed 5 August, 2018).

[B7] Ishikawa Y. Miyakoshi N. Hongo M. Kasukawa Y. Kudo D. Shimada Y. (2017). Relationships among spinal mobility and sagittal alignment of spine and lower extremity to quality of life and risk of falls. Gait Posture 53, 98–103. 10.1016/j.gaitpost.2017.01.011 28126694

[B8] Kasukawa Y. Miyakoshi N. Hongo M. Ishikawa Y. Noguchi H. Kamo K.. (2010). Relationships between falls, spinal curvature, spinal mobility and back extensor strength in elderly people. J. Bone Miner. Metab. 28, 82–87. 10.1007/s00774-009-0107-1 19690799

[B9] Lan C. Lai J. S. Chen S. Y. Wong M. K. (1998). 12-month Tai Chi training in the elderly: its effect on health fitness. Med. Sci. Sports Exerc. 30, 345–351. 10.1097/00005768-199803000-00003 9526879

[B10] Liu T. Chan A. W. K. Liu Y. H. Taylor-Piliae R. E. (2018). Effects of Tai Chi-based cardiac rehabilitation on aerobic endurance, psychosocial well-being, and cardiovascular risk reduction among patients with coronary heart disease: a systematic review and meta-analysis. Eur. J. Cardiovasc. Nurs. 17, 368–383. 10.1177/1474515117749592 29256626

[B11] Schenkman M. Laub K. C. Kuchibhatla M. Ray L. Shinberg M. (1997). Measures of shoulder protraction and thoracolumbar rotation. J. Orthop. Sports Phys. Ther. 25, 329–335. 10.2519/jospt.1997.25.5.329 9130150

[B12] Wang L. Wu K. Chen X. Liu Q. (2019). The effects of Tai Chi on lung function, exercise capacity and health related quality of life for patients with chronic obstructive pulmonary disease: a pilot study. Heart Lung Circ. 8, 1206–1212. 10.1016/j.hlc.2018.05.204 30166260

[B13] Wehner Ch Blank C. Arvandi M. Wehner Ca Schobersberger W. (2021). The effect of Tai Chi on muscle strength, physical endurance, postural balance and flexibility - a systematic review and meta-analysis. BMJ Open Sport Exerc. Med. 7, e000817. 10.1136/bmjsem-2020-000817 33614126 PMC7871341

[B14] Wen C. Cao X. Zhang Y. Crouter S. E. Wozencroft A. J. Zhang S. (2018). Knee biomechanics of selected knee-unfriendly movement elements in 42-form Tai Chi. Int. J. Perform. Anal. Sport 18, 1050–1066. 10.1080/24748668.2018.1553093

[B15] Zhang J. G. Ishikawa-Takata K. Yamazaki H. Morita T. Ohta T. (2006). The effects of Tai Chi Chuan on physiological function and fear of falling in the less robust elderly: an intervention study for preventing falls. Arch. Gerontol. Geriatr. 42, 107–116. 10.1016/j.archger.2005.06.007 16125805

[B16] Zhou Z. Zhou R. Li K. Zhu Y. Zhang Z. Luo Y.. (2019). Effects of tai chi on physiology, balance and quality of life in patients with type 2 diabetes: a systematic review and meta-analysis. J. Rehabil. Med. 51, 405–417. 10.2340/16501977-2555 30968941

