# Supplementary material for: Thoracolumbar Rotation During Tai Chi Movements—A Biomechanical Analysis of the Entire Peking Style Routine
Source: Front Sports Act Living. 2022 Feb 2;4:834355. doi: 10.3389/fspor.2022.834355 (PMC8849243; doi:10.3389/fspor.2022.834355)
Supplement: Supplementary file 1 [file Table_1.DOCX]

Supplementary Material

Table 1. Range of motion of thoracolumbar rotation (HS)

| Movement name | Athlete | | | | | | | | |
| --- | --- | --- | --- | --- | --- | --- | --- | --- | --- |
|  | 1 | | 2 | 3 | 4 | 5 | 6 | 7 | 8 |
| Beginning | | 3.83 | 6.43 | 6.57 | 3.38 | 4.80 | 4.93 | 3.75 | 3.88 |
| Part Wild Horse‘s Mane (left) | | 11.95 | 15.63 | 15.33 | 9.98 | 15.49 | 9.42 | 22.01 | 17.65 |
| Part Wild Horse‘s Mane (right) | | 13.17 | 12.57 | 11.33 | 9.04 | 14.78 | 10.60 | 23.98 | 20.53 |
| White Crane Spreads Its Wings | | 45.12 | 19.23 | 25.25 | 38.32 | 16.57 | 20.36 | 19.41 | 31.70 |
| Brush Knee, Step Forward (left) | | 74.49 | 57.13 | 60.33 | 57.55 | 63.55 | 37.26 | 47.87 | 42.14 |
| Brush Knee, Step Forward (right) | | 36.62 | 29.28 | 35.63 | 29.06 | 34.31 | 28.08 | 42.47 | 44.32 |
| Playing the Lute | | 29.52 | 22.16 | 18.51 | 23.14 | 17.83 | 12.14 | 17.90 | 25.94 |
| Step Back, Repulse Monkey (left) | | 29.82 | 26.88 | 50.05 | 36.24 | 24.01 | 24.98 | 35.74 | 32.58 |
| Step Back, Repulse Monkey (right) | | 23.69 | 27.15 | 35.46 | 25.64 | 33.58 | 14.92 | 21.01 | 26.32 |
| Grasp the Sparrow‘s Tail (left) | | 29.97 | 26.11 | 37.06 | 31.58 | 28.16 | 17.95 | 34.14 | 49.23 |
| Grasp the Sparrow‘s Tail (right) | | 34.31 | 23.21 | 27.77 | 25.90 | 27.35 | 27.16 | 53.36 | 48.28 |
| Single Whip | | 9.95 | 11.11 | 8.79 | 21.67 | 10.17 | 12.98 | 9.86 | 17.24 |
| Wave Hand in the Clouds | | 46.80 | 51.77 | 42.83 | 47.70 | 49.59 | 48.18 | 42.55 | 30.98 |
| High Pat on Horse | | 39.27 | 39.41 | 46.80 | 39.10 | 35.31 | 29.87 | 35.35 | 41.10 |
| Right Heel Kick | | 14.01 | 13.87 | 22.49 | 15.85 | 13.53 | 18.57 | 5.57 | 18.68 |
| Strike to Ears with Both Fists | | 33.51 | 34.89 | 24.63 | 34.91 | 27.34 | 25.39 | 30.59 | 31.69 |
| Left Heel Kick | | 39.48 | 17.97 | 18.14 | 24.89 | 16.99 | 42.01 | 25.35 | 18.75 |
| Lower Body, Stand on One Leg (left) | | 33.88 | 33.93 | 32.21 | 34.13 | 27.01 | 31.84 | 31.75 | 21.10 |
| Lower Body, Stand on One Leg (right) | | 37.18 | 30.09 | 34.67 | 28.90 | 28.82 | 38.19 | 30.87 | 24.72 |
| Fair Lady Weaves with Shuttle (right and left) | | 57.14 | 55.13 | 19.66 | 48.21 | 41.29 | 42.73 | 37.69 | 50.92 |
| Fair Lady Weaves with Shuttle (right and left) | | 59.24 | 58.42 | 30.87 | 63.35 | 49.53 | 47.27 | 53.52 | 57.74 |
| Pick Up the Needle from the Sea Bottom | | 49.77 | 48.45 | 33.02 | 60.68 | 51.70 | 43.69 | 45.78 | 52.65 |
| Fan Back | | 15.42 | 3.87 | 16.36 | 4.22 | 15.37 | 10.30 | 7.26 | 5.77 |
| Turn Body, Deflect, Parry, and Punch | | 33.70 | 51.59 | 20.97 | 31.59 | 20.35 | 28.61 | 36.91 | 32.02 |
| Seal Tightly | | 21.37 | 23.96 | 5.98 | 10.55 | 14.47 | 7.22 | 8.93 | 12.65 |
| Cross Hands | | 19.47 | 11.48 | 7.77 | 5.76 | 18.90 | 11.46 | 2.16 | 11.36 |
| Closing | | 7.35 | 4.82 | 14.01 | 16.77 | 16.26 | 11.77 | 17.14 | 8.85 |

Range of motion of the health sport version in degrees.

Table 2. Range of motion of thoracolumbar rotation (CV)

| Movement name | Athlete | | | | | | | | |
| --- | --- | --- | --- | --- | --- | --- | --- | --- | --- |
|  | 1 | | 2 | 3 | 4 | 5 | 6 | 7 | 8 |
| Beginning | | 5.27 | 5.98 | 7.77 | 3.75 | 6.37 | 4.94 | 4.21 | 5.46 |
| Part Wild Horse‘s Mane (left) | | 26.51 | 15.92 | 29.98 | 13.09 | 18.58 | 7.74 | 26.03 | 32.28 |
| Part Wild Horse‘s Mane (right) | | 35.86 | 22.28 | 24.91 | 11.51 | 11.87 | 4.92 | 31.85 | 26.67 |
| White Crane Spreads Its Wings | | 64.28 | 51.65 | 28.22 | 48.13 | 29.95 | 17.24 | 38.09 | 37.48 |
| Brush Knee, Step Forward (left) | | 65.77 | 42.86 | 50.00 | 60.98 | 53.06 | 46.67 | 50.95 | 44.48 |
| Brush Knee, Step Forward (right) | | 58.54 | 41.11 | 59.19 | 39.82 | 36.74 | 39.01 | 42.62 | 55.74 |
| Playing the Lute | | 25.01 | 42.25 | 29.45 | 23.38 | 25.22 | 19.93 | 21.94 | 29.15 |
| Step Back, Repulse Monkey (left) | | 31.84 | 24.74 | 42.88 | 32.77 | 26.18 | 28.89 | 37.50 | 29.72 |
| Step Back, Repulse Monkey (right) | | 29.70 | 24.26 | 38.52 | 33.38 | 19.20 | 25.19 | 18.82 | 31.28 |
| Grasp the Sparrow‘s Tail (left) | | 55.54 | 48.91 | 46.62 | 40.33 | 33.21 | 37.64 | 39.85 | 55.12 |
| Grasp the Sparrow‘s Tail (right) | | 61.64 | 44.06 | 46.54 | 35.19 | 24.79 | 34.27 | 64.83 | 52.05 |
| Single Whip | | 16.93 | 13.72 | 15.19 | 21.66 | 19.26 | 17.40 | 47.59 | 19.89 |
| Wave Hand in the Clouds | | 55.07 | 47.52 | 33.91 | 49.57 | 46.36 | 40.41 | 43.75 | 39.96 |
| High Pat on Horse | | 45.92 | 45.33 | 55.11 | 40.30 | 34.76 | 32.41 | 45.63 | 41.43 |
| Right Heel Kick | | 28.07 | 39.74 | 26.18 | 23.05 | 26.21 | 21.42 | 11.61 | 22.78 |
| Strike to Ears with Both Fists | | 48.12 | 55.56 | 36.46 | 38.30 | 46.63 | 39.48 | 31.07 | 46.26 |
| Left Heel Kick | | 53.27 | 34.03 | 21.69 | 36.41 | 35.51 | 37.92 | 24.72 | 26.06 |
| Lower Body, Stand on One Leg (left) | | 38.24 | 36.19 | 25.38 | 38.17 | 32.81 | 37.66 | 33.59 | 34.56 |
| Lower Body, Stand on One Leg (right) | | 58.02 | 38.88 | 33.28 | 35.27 | 28.46 | 32.50 | 23.90 | 39.63 |
| Fair Lady Weaves with Shuttle (right and left) | | 78.91 | 52.89 | 48.86 | 58.38 | 58.66 | 53.58 | 57.22 | 65.54 |
| Fair Lady Weaves with Shuttle (right and left) | | 82.74 | 76.88 | 69.26 | 86.07 | 66.81 | 55.71 | 65.13 | 75.14 |
| Pick Up the Needle from the Sea Bottom | | 58.93 | 62.71 | 35.14 | 67.05 | 64.05 | 61.33 | 55.20 | 62.34 |
| Fan Back | | 22.22 | 24.76 | 25.88 | 16.37 | 22.40 | 13.61 | 7.13 | 12.64 |
| Turn Body, Deflect, Parry, and Punch | | 34.41 | 52.14 | 37.11 | 33.83 | 21.01 | 29.77 | 36.57 | 37.73 |
| Seal Tightly | | 33.08 | 25.88 | 14.25 | 14.05 | 18.87 | 5.23 | 12.56 | 15.62 |
| Cross Hands | | 28.31 | 43.23 | 13.62 | 4.07 | 16.17 | 11.43 | 6.10 | 22.34 |
| Closing | | 15.08 | 5.73 | 14.63 | 20.59 | 12.26 | 9.20 | 29.11 | 13.88 |

Range of motion of the competition version in degrees.
